# Interleukin-23 Involved in Fibrotic Autoimmune Diseases: New Discoveries

**DOI:** 10.3390/jcm12175699

**Published:** 2023-09-01

**Authors:** Margherita Sisto, Sabrina Lisi

**Affiliations:** Department of Translational Biomedicine and Neuroscience (DiBraiN), Section of Human Anatomy and Histology, University of Bari “Aldo Moro”, 70123 Bari, Italy; sabrina.lisi@uniba.it

**Keywords:** IL-23, autoimmunity, inflammation, fibrosis

## Abstract

Interleukin (IL)-23 is a central pro-inflammatory cytokine with a broad range of effects on immune responses. IL-23 is pathologically linked to the induction of the production of the pro-inflammatory cytokines IL-17 and IL-22, which stimulate the differentiation and proliferation of T helper type 17 (Th17) cells. Recent discoveries suggest a potential pro-fibrotic role for IL-23 in the development of chronic inflammatory autoimmune diseases characterized by intense fibrosis. In this review, we summarized the biological features of IL-23 and gathered recent research on the role of IL-23 in fibrotic autoimmune conditions, which could provide a theoretical basis for clinical targeting and drug development.

## 1. Introduction

Multiple autoimmune diseases are a group of clinically heterogeneous conditions that show common inflammatory signaling pathways arising from aberrant immune responses [1]. Some of these disorders are characterized by intense and severe fibrotic processes as the result of a complex interplay between different immune cell types following persistent inflammatory activity [2,3]. Autoimmune diseases, traditionally characterized by chronic inflammation, present tissue damage that often evolves towards fibrosis. This represents a serious clinical problem because it causes organ failure. The fibrotic evolution of autoimmune diseases presents an excessive release of pro-fibrotic factors as a result of the activation of molecular cascades depending on chronic inflammation. In recent years, researchers’ efforts have focused on identifying molecular bridges that can connect the various fibrotic pathways identified so far [2,3]. Several cytokines have been well studied for their ability to generate inflammatory loops through positive feedback mechanisms [4]. Breaking these loops through cytokine neutralization has proved that inflammation attenuates and ameliorates the disease [5]. One of these cytokines is interleukin (IL)-23, a multifunctional pro-inflammatory cytokine that is involved in a variety of biological processes [6]. Although the role of IL-23 in the immune response during bacterial and viral infections has recently been evaluated, demonstrating its central role [7], its deregulation has been demonstrated to aggravate chronic inflammatory status, contributing to the development of autoimmune diseases [8,9]. As shown by several authors, IL-23 is involved in the pathogenesis of several autoimmune diseases [10,11,12,13]. The importance of IL-23 implicated in the evolution of autoimmune pathologies was demonstrated by analyzing the susceptibility of IL-12- or IL-23-deficient mice [10,11]. Indeed, mice that have a deletion of IL-23 were protected from disease in several experimental models of autoimmunity. Importantly, treatment of mice with anti-IL-23 prevents the development of autoimmune conditions [12].

In this review, we provide an overview of the most recent discoveries, focusing on the role of IL-23 in fibrotic pathways and its role in the pathogenesis of inflammatory autoimmune diseases characterized by fibrotic evolution.

## 2. Structure of IL-23 and Its Receptor

**Figure 1:**
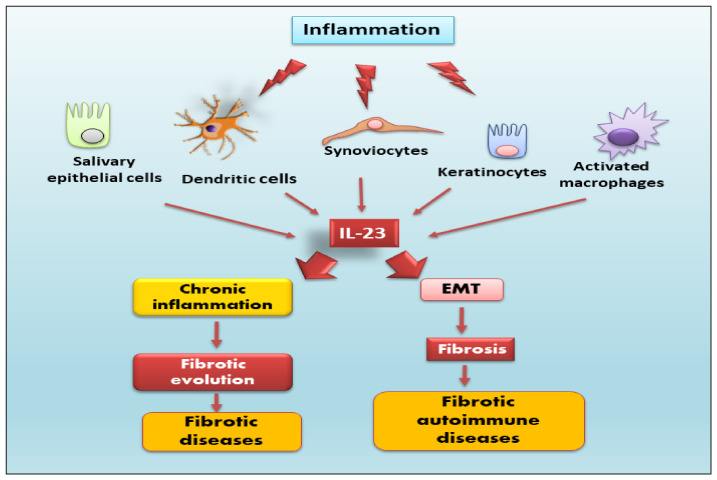
IL-23 is expressed and secreted by activated macrophages and dendritic cells located in different tissues and by non-immune cells, such as keratinocytes, synoviocytes, and salivary gland epithelial cells. IL-23, through several signaling pathways, provokes, on one side, the activation of the epithelial–mesenchymal transition (EMT) mechanism, inducing fibrotic processes in autoimmune diseases, and, on the other side, chronic inflammation that determines severe fibrotic evolution in several diseases.

IL-23 is a heterodimeric member with pro-inflammatory characteristics that belongs to the special IL-12 family, and it is constituted of two different subunits: p19 and p40 [14]. The p40 subunit is a glycosylated type I soluble protein that has a molecular weight of 34.7 kDa and is positioned on the 11q1.3 chromosome [15,16]. The p19 subunit is a non-glycosylated protein with a molecular weight of 18.7 kDa located on chromosome 12q13.2 [8]. Both subunits are linked by a disulphide bond, and they are attached only if they are synthesized in the same cell [8,14]. Specifically, IL-23 is expressed and secreted by activated macrophages and dendritic cells located in several tissues, such as the skin, intestinal mucosa, joints, and lungs (Figure 1).

**Figure 2:**
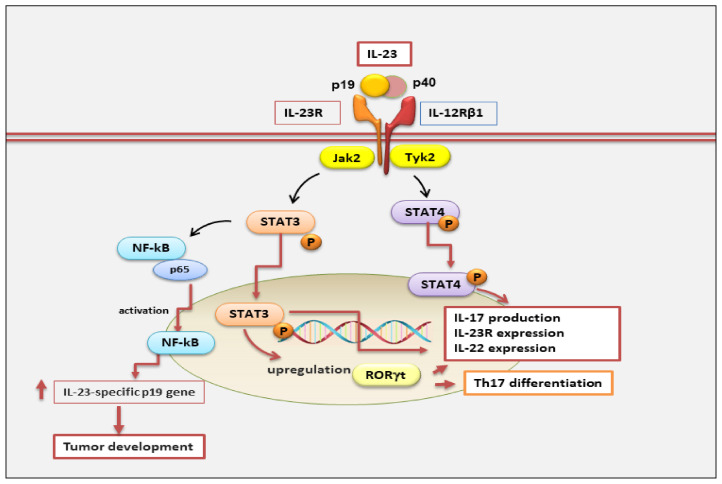
IL-23 is a heterodimeric cytokine composed of p40 and p19 subunits. It binds to its IL-23 receptor complex, composed of IL-12Rb1 and IL-23R subunits, which are linked to Jak2 and Tyk2, respectively. Active phosphorylated Jak2/Tyk2 leads to phosphorylation of STAT3 and STAT4. Phospho-STAT3 and phospho-STAT4 translocate into the nucleus, inducing transcription of cytokines, such as IL-17 and IL-22, and differentiation of T helper 17. STAT3 plus RORγt cooperate to increase IL23R, IL-17, and IL-22 expression and to stabilize the Th17 phenotype. The binding of IL-23 to its receptor through NF-κB/p65 activation leads to tumor progression. Jak2, Janus kinase 2; RORγt, RAR-related orphan receptor gamma; Tyk2, tyrosine kinase 2.

Interestingly, it is also secreted by non-immune cells, such as keratinocytes, synoviocytes, and salivary gland epithelial cells [17,18,19]. The IL-23 signaling pathway occurs through a link with its receptor. IL-23 receptor (IL-23R) is a heterodimeric structure that consists of two subunits: a heterodimer with the IL-12Rβ1 subunit and its unique IL-23R subunit, positioned on human chromosome 19 and encoding the gene that constitutes the IL-12Rβ1 subunit, and on human chromosome 1, encoding the gene that forms the IL-23R subunit [20]. The IL-12Rβ1 subunit is principally expressed on T cells, monocytes/macrophages, natural killer T cells, and dendritic cells [7,21], with minor expression on B cells and lymphoid cells [22] (Figure 2).

## 3. Regulation of IL-23 Signaling

Since its discovery, IL-23 has received widespread attention, and although it has a similar structure to IL-12, its role is totally different. Indeed, in spite of the protective function addressed by IL-23 against bacterial, fungal, and viral infections, extensive knowledge sustains the contribution of its alteration in triggering chronic inflammation and autoimmunity, providing a solid substrate for the development of several autoimmune diseases, like psoriasis, systemic lupus erythematosus (SLE), rheumatoid arthritis (RA), Sjögren’s syndrome (SS), and multiple sclerosis (MS) [8,11,23,24].

Currently, the IL-23 signaling pathway remains largely uncharacterized. IL-23 involves the activation of members of the Janus family of tyrosine kinases (JAKs), their downstream factors, and the signal transducers and activators of transcription (STATs) family [25]. In particular, IL-23, through binding to its IL-23 receptor, provokes phosphorylation and activation of JAK/STAT signaling molecules (Jak2, Tyk2), promoting STAT3 and STAT4 phosphorylation and activation [25]. Subsequently, active STAT3 up-regulates the expression of the transcription factor RORγt, which is critical for IL-17 production [26]. Indeed, STAT3 plus RORγt cooperate to facilitate a positive loop that increases IL23R, IL-17, and IL-22 expression and that stabilizes the Th17 phenotype. Thus, STAT4 phosphorylation and activation promote upregulation of IL23R, IL-17, and IL-22 expression and the increase of Th17 [26]. However, other IL-23-regulated mechanisms are involved in the evolution of disease. For example, STAT3 activation is not restricted to IL-23 because other cytokines, such as IL-6, IL-21, IL-10, or IL-27, induce STAT3 activation without triggering adverse effects; rather, in some cases, it exerts anti-inflammatory events [25] (Figure 2).

To date, multiple cytokines play complex roles in IL-23 regulation. IL-23 was shown to be upregulated in fibroblast-like synoviocytes in response to IL-1β and Tumor Necrosis Factor (TNF)-α [27,28], while TNF-α receptor 1 can decrease IL-23 expression by downregulating subunit p40 [29]. Likewise, the cytokine IL-10, which has an anti-inflammatory role, can also diminish IL-23 expression [30].

A mounting number of studies have evidenced [23,31] that the key role of IL-23 is to drive the differentiation of T CD4+ naive cells into Th17 cells [32,33]. This leads to enhanced IL-17 production, considered a crucial player in the pathogenesis of inflammatory and autoimmune diseases [34,35]. Thus, IL-23/IL-17 axis activation leads to the onset of several inflammatory autoimmune diseases, and results obtained from experimental mouse models confirm the crucial role of the IL-23/IL-17 axis in the pathogenesis of various autoimmune conditions, such as arthritis [36]. Consequently, suppressing the trigger of the IL-23/IL-17 axis improves the inflammatory condition and is considered a promising therapeutic approach in patients with these disorders [34].

Recent advances have reported that IL-23 induction can also occur through Toll-like receptor (TLR, TLRs) signaling. It has been demonstrated that Theiler’s murine encephalomyelitis virus (TMEV), which leads to infection of central nervous system microglia and macrophages in mice, provoking a disorder similar to MS in humans, stimulates the expression of IL-23 via binding to TLR3 and TLR7, contributing to the development of experimental autoimmune MS [37]. Additionally, other research findings have reported that IL-23 induction can also occur through TLR9 or cooperatively with other TLRs [37,38].

More recently, IL-23 has been demonstrated to be regulated during tumor-promoting development and to have protumor immunity [39]. An interesting study has demonstrated that Stat3 induces expression of IL-23, which is mainly secreted by macrophages in the tumor microenvironment via transcriptional activation of the IL-23/p19 gene and through NF-κB/p65 activation, promoting tumor development. In contrast, Stat3 also inhibits NF-kappaB/c-Rel-dependent IL-12/p35 gene expression in cancer-linked dendritic cells. Furthermore, tumor-associated regulatory T cells (Tregs) express the IL-23 receptor, which stimulates the expression of Stat3 in dendritic cells, leading to upregulation of the Treg-specific transcription factor Foxp3 and the immunosuppressive cytokine IL-10. These results demonstrate that Stat3 induces IL-23-mediated tumorigenes [40]. However, findings of IL-23′s antitumorigenic and antimetastatic characteristics demonstrated that IL-23 induced long-term regression of tumors similar to that of IL-12-transduced cancers. Other studies have also shown that CD40 ligand expression on lung tumor cells activates the immune response, determining increased transcription of p19 and p40 subunits and influencing the regression of the tumors [41,42].

It has been suggested, based largely on in vitro observations, that IL-23 stimulation increases the number of already-differentiated Th-17 cells and maintains IL-17 production from Th17 cells. For example, the addition of IL-23 during the culture of activated or memory T cells results in an increase in their proliferation and the frequency of IL-17+ T cells produced [43]; IL-23 is also required during restimulation of Th17 cells (i.e., cells previously stimulated with TGF-β and IL-6 to maintain IL-17 production from the Th-17 cells) [44]. Similarly, it has been suggested that IL-23 may stabilize the phenotype of Th17 cells through mechanisms dependent on the transcription factor STAT3 [45,46]. Two other cytokines thought to be involved in Th17 differentiation, IL-6 and IL-21, also share the STAT3-dependent signaling pathway with IL-23.

Emerging evidence has highlighted that IL-23 can be considered a survival factor for Th17 cells [35]. These data are confirmed by the observation of reduced frequencies of Th17 cells in mice lacking the IL-23 gene [43].

Finally, several findings have highlighted the profibrogenic role of IL-23 as a promoter of the epithelial–mesenchymal transition (EMT) process, an aberrant pro-fibrotic response to repetitive injury of epithelia, and the acquisition of a mesenchymal phenotype. High levels of IL-23 have been found in some chronic inflammatory autoimmune disorders characterized by fibrosis [13,14,28].

## 4. Role of IL-23 in the Fibrotic Process

IL-23 is known to mediate inflammatory conditions through the induction of Th17 cells, which produce the pro-inflammatory IL-17 [12]. Because the prevailing hypothesis is that the fibrotic evolution of diseases is preceded by chronic inflammation, therapeutic strategies blocking IL-23 were suggested as a promising approach, though the specific role of IL-23 in fibrosis needs to be clarified. This section summarizes the most recent findings regarding the role of IL-23 in fibrotic diseases.

### 4.1. Idiopathic Pulmonary Fibrosis

Idiopathic pulmonary fibrosis (IPF) is characterized by chronic progressive fibrosis of the lung [47]. In pulmonary fibrosis, in vitro experiments have demonstrated that treatment with an anti-interleukin-23-specific antibody attenuated airway inflammation and reduced fibrosis by blocking interleukin-17A and -22 release. The role of IL-23 in the pathophysiology of respiratory diseases is attracting increasing attention from researchers. An important role of IL-23 in the pathological features of pulmonary emphysema was demonstrated [48], and the involvement of IL-23 in the pathogenesis of a lipopolysaccharide-induced animal model of acute respiratory distress syndrome was also demonstrated [49]. Also, in the overall accepted bleomycin or IL-1β-induced pulmonary fibrosis, both IL-23 and IL-17A play a significant role in this context. The data collected suggest that IL-23 and IL-17A are essential for fibrosis pathway activation [50]. Moreover, the data seem to point towards a priority role for IL-23, which would also be responsible for the consequent secretion of IL-17 and the acute exacerbation of pulmonary fibrosis; in fact, by blocking the secretion of IL-23, the release of IL-17 is also decreased [50]. In addition, IL-22, IL-23, and IL-17 were significantly increased in the serum of lung cancer patients associated with IPF; the levels of these cytokines found in the serum show such significant values that they represent a parameter used to discriminate between lung cancer patients and the lung-cancer-associated IPF group; finally, the expression of IL-22, IL-23, and IL-17 was positively correlated with the degree of differentiation and tumor metastasis [51].

### 4.2. Inflammatory Bowel Diseases

Idiopathic inflammatory bowel diseases (IBD) represent chronic inflammatory diseases of the gastro-intestinal tract characterized by a strong inflammatory component, often on an autoimmune basis, against the intestinal microbiome; the main diseases that fall into this group are Crohn’s disease and ulcerative colitis [52,53]. Intestinal fibrosis is a severe complication of IBD. With the establishment of a fibrotic evolution, the intestinal wall undergoes substantial structural modifications that lead to stiffness and a reduction in caliber. These phenomena, in the long run, seriously compromise the quality of life of patients. Chronic inflammation is certainly a factor that predisposes to fibrotic evolution. Furthermore, currently, there are no drugs that have shown efficacy in blocking intestinal fibrosis or improving the pathological condition. For these reasons, surgical treatment remains the only intervention strategy in cases of intestinal fibrosis and stenosis. Interleukin (IL)-12 and IL-23, with their structural similarities, are important cytokines in the pathogenesis of IBD. Recent data reported the efficacy of p40 peptide-based vaccines on intestinal inflammation in a colitis model made in a laboratory [54]. The results demonstrate that the administration of the vaccine reduces the clinical symptoms, slows down the fibrotic process with a significant attenuation of the inflammatory parameters, and reduces the release of pro-inflammatory cytokines, leading to an improvement in intestinal conditions in the mouse model used. In addition, in the intestinal lamina propria and in the local lymph nodes, a high ratio of Treg/Th1 and Treg/Th17 cells was detected [54]; furthermore, in CD11c+ dendritic cells, the vaccine stimulates the release of IL-10, which is critical to controlling small intestinal immune homeostasis by limiting the reactivation of local memory T cells and so attenuating excessive immune responses [54]. These data confirm a key role for IL-23 in the modulation of the activation of Th17 cells involved in fibrosis pathway activation.

### 4.3. Liver Fibrosis

Non-alcoholic fatty liver disease (NAFLD) is a condition in which the liver has an excess of fat deposits [55]. Two types of NAFLD are non-alcoholic fatty liver (NAFL) and non-alcoholic steatohepatitis (NASH). People generally develop one form of NAFLD, but a good percentage appear to be predisposed to developing the second form in the long run as well. NAFL and NASH are constantly increasing around the world [56]. NASH is currently considered the progressive step of NAFLD. It is described in liver steatosis, inflammation, and fibrosis with different severity [57]. A predominant role in the inflammatory condition characterizing NASH is played by Th17 cells, which express high levels of IL-17 in response to IL-23. A vital transcription factor for Th17 is the retinoic acid receptor-related orphan receptor γt (RORγt), whose enhanced expression and nuclear translocation are correlated with IL-23-dependent phosphorylation and dimerization of the signal transducer and activator of transcription 3 (STAT3) [58]. Because no targeted therapies have been identified for NAFLD/NASH, any factor that attenuates the inflammatory process and the related fibrotic process is evaluated experimentally in order to test its efficacy in these complex diseases [59]. In this respect, it is useful for scientists to hold in high regard the fact that hepatic IL-17-producing cells promote liver inflammation and dysfunction [60]. However, the genetic analysis carried out in subjects at risk of developing the NASH disease but who do not yet show clinical signs has not yet given certain data regarding a possible alteration of the gene that synthesizes IL-23, and the candidacy of the IL-17/IL-23 axis in NASH is yet to be fully established. Experimental data obtained using a mouse model with a double mutation in the IL-23 gene have, however, shown that protection against chronic inflammation and fibrotic development is moderate but not total, and this seems to suggest that although the IL-17/IL-23 axis is involved, it is probably related to the activation of other inflammatory pathways, and targeting IL-23 signaling activation may not be the only therapeutic approach for NASH [61].

## 5. Novel Role of IL-23 in Autoimmune Fibrotic Diseases

IL-23 is a factor involved in the development of autoimmune diseases, such as multiple sclerosis (MS), rheumatoid arthritis (RA), and systemic lupus erythematosus; it carries out its activity by stimulating and activating the pathogenic Th17 cells. Therefore, IL-23 is a potential target for modulating autoimmune responses and pathogenic Th17 cell effects. Recently developed clinical trials have shown the beneficial effects of blocking the IL-23/Th17 pathway in chronic inflammatory autoimmune diseases characterized by fibrotic damage to the organs. Here, we report the most recent and pioneering discoveries in this field.

### 5.1. Rheumatoid Arthritis

**Figure 3:**
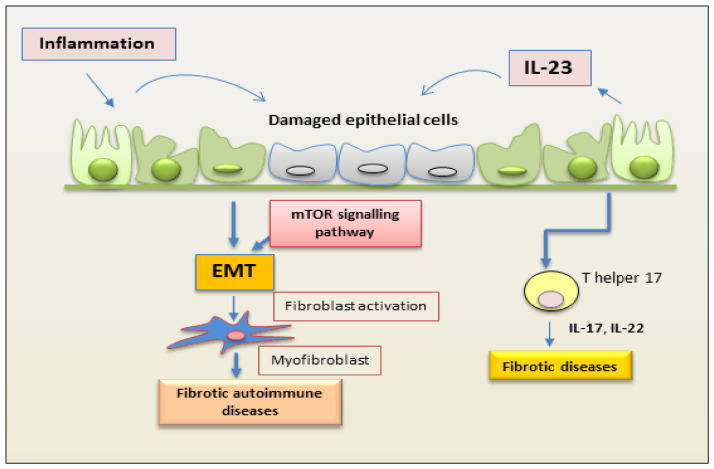
Schematic representation of a proposed mechanism by which IL-23 mediates EMT in epithelial cells. Inflammation promotes repetitive injury to epithelial cells that leads to the secretion of IL-23, inducing the EMT process. Injured cells start transforming into mesenchymal cells, and IL-23 amplifies the EMT process. On binding to its receptor, IL-23 activates the kinase mTOR and promotes the expression of mesenchymal markers. This process induces fibrosis in autoimmune diseases. (EMT, epithelial–mesenchymal transition; mTOR, mammalian target of rapamycin).

The role of IL-23 in RA has been extensively studied in a co-morbidity that affects approximately 15% of RA patients that is termed RA interstitial lung disease (RA-ILD) [62]. How much an excessive or altered immune response mediated by Th17 activation is involved in this pathology remains to be demonstrated. Recently, insight on the direct role of IL-23 in lung fibrosis was obtained by experimentally investigating the responsiveness of lung fibroblasts to IL-23 stimulation. The induction of CCR2 expression that regulates monocyte chemotaxis and the increased fibroblast migration suggest a direct role for IL-23 in fibrotic lung disease associated with a Th17-biased immune response [63]. In this context, a process strictly correlated with fibrotic evolution [2,3] plays a role, represented by EMT, which seems to be activated in the lung by chronic inflammatory stimuli and tissue damage. The EMT process creates an environment that facilitates fibrosis when alveolar epithelial cells are injured. In this scenario, IL-23 exerts its profibrogenic role on somatic alveolar type I (ATI) epithelial cells [63]. Primary ATI cells, after prolonged culture on rigid culture dishes, clearly show signs of a gradual transformation towards a mesenchymal phenotype characterized by the loss of epithelial proteins, such as caveolin-1, and by a reorganization of the F-actin cytoskeleton, indicating the initiation of the EMT process. IL-23 appears to be actively involved in this process because the mesenchymal transformation process is accelerated by in vitro stimulation with this cytokine, which results in the loss of the epithelial marker caveolin-1 and increased expression of mesenchymal markers, such as α-smooth muscle actin (α-SMA) and collagen I/III protein. Furthermore, IL-23 significantly promotes cell migration and regulates apoptotic resistance in IL-23-transitioning-treated ATI cells [63]. IL-23-induced EMT seems to be activated and regulated by the Target of Rapamycin (mTOR)/S6 signaling pathway, which has already been demonstrated to be the pathway involved in the pro-fibrotic activity of IL-23 [64]. The hypothesis of an involvement of IL-23 in the pathogenesis of RA-ILD exerted by promoting mTOR/S6 signaling-dependent EMT in alveolar epithelial cells was supported by transcriptional sequencing analysis of human lung fibrosis biopsy tissue [63], which detected a significant increase in IL-23 mRNA expression in RA-ILD lung sections positively correlated with transitioning ATI epithelial cell [63] (Figure 3).

### 5.2. Crohn’s Disease

Intestinal fibrosis is an important complication of Crohn’s disease (CD) characterized by exaggerated proliferation of myofibroblasts and increased deposition of collagen in response to prolonged injury or chronic inflammation typical of IBD [65,66]. The mechanism underlying this hyperproliferation of myofibroblasts in CD seems to be linked, as in other pathological conditions mentioned above, to an involvement of mTOR. Experimental data report that the inhibition of mTOR determines a decreased production of IL-23, which, in turn, negatively regulates IL-22 expression and determines an improvement in the general conditions of the mouse model and a slowdown in the fibrotic evolution [66]. This inhibition of IL-23 expression is associated with elevated autophagy activity in intestinal Cx3cr1+ mononuclear phagocytes. This result paves the way to identifying a new molecular pathway that can explain the intestinal fibrotic progression in CD. The autophagy gene Atg7 knockdown determines, in fact, increased IL-23 expression and, consequently, induces the release of IL-22, triggering the fibrotic molecular events’ activation [65]. This evidence suggests, once again, a correlation between the production of IL-23 and IL-22 and identifies a new activation pathway of the fibrotic process that originates in Cx3cr1+ mononuclear phagocytes, in which the mTOR/autophagy pathway regulates the IL-23/IL-22 axis-dependent fibrosis. The synergistic action performed by IL-23 and IL-22 is elucidated and confirmed by the fact that the double inhibition of the release of both cytokines determines a decrease in all the parameters characterizing fibrosis [65] (Figure 3).

### 5.3. Autoimmune Myocarditis

Recent observations have correlated IL-23 levels with the regulation of T cells’ function in autoimmune myocarditis, thus confirming the role of IL-23 in the regulation of inflammatory processes [67]. Using mice mutated for the IL-23 gene and unable to produce a functional interleukin, IL23a−/− mice, it was demonstrated that IL-23 is necessary for the induction of cardiac inflammation in experimentally induced autoimmune myocarditis (EAM) [68]. EAM has been considered a disease characterized by the altered function of CD4+ T cells [67]. In addition, transfection experiments demonstrated that IL-23 was able to restore pathogenicity to CD4+ T cells lacking the IL-23 gene. These results support the hypothesis of a direct involvement of IL-23 in the autoimmune reactions of the heart, which could occur either thanks to the action of IL-23 on the activity of T helper cells or by inducing the secretion by the T helpers of a combination of cytokines capable of triggering autoimmune responses [69]. Both hypotheses seem to be valid and have found scientific evidence; in fact, continuous stimulation through IL-23 is necessary to determine the production of IL-17A by T helper lymphocytes. On the other hand, even a temporary stimulation by IL-23 seems to be sufficient to determine a pathogenic activation of the T helper. Confirming this, the lack of IL-23 does not compromise the establishment of an EAM condition when the T helpers have become autoreactive [68]. Based on this evidence, because high levels of IL-23 were found in patients with autoimmune myocarditis, which proceeded to stimulate T helper cells, determining an increased production and release of IL-17A, a therapy against IL-23 could be effective in blocking or delaying the fibrotic progression of the disease [68]. Moreover, elevated fibrosis and impaired heart function were detected in IL-23−/− mice but not in mice lacking the IL-12 gene at the chronic stage of the disease; this underlines the importance of IL-23-dependent T cell activation in the resolution phase of the acute stage of autoimmune myocarditis.

### 5.4. Sjögren’s Syndrome

Sjögren’s syndrome (SS) is an autoimmune disease characterized by a chronic inflammatory response that causes a morphological and functional alteration of the exocrine glands, in particular the salivary and lacrimal glands, and this seriously compromises the quality of life of these patients [2,3]. “Primary” SS is defined as a standalone entity occurring in the absence of another systemic autoimmune disease, whereas “secondary” disease is associated with the presence of other autoimmune conditions, such as RA, SLE, or systemic sclerosis (SSc). Currently, the data relating to the involvement of IL-23 in the pathogenesis of SS are still few, although very promising. Previous research revealed that IL-17/IL-23 expression was increased in mouse models of SS, highlighting that Th17 participates in lymphocytic infiltration of salivary glands and leads to lesion formation [70,71]. Another experimental study demonstrated that both protein and mRNA levels of IL-22, IL-23, and IL-17 were enhanced in the peripheral blood of patients affected by SS [72], assuming that the IL-23/IL-17/IL-22 axis could be one of the key mediators in the pathogenesis of primary SS [70,72,73,74]. Interestingly, in the absence of certain evidence of primary SS, the immunohistochemical detection of IL-17/IL-23 would classify these patients as involved in a Th17 reaction and lead to the selection of patients to be referred for subsequent periodic diagnostic screening. Based on this assumption, the use of IL-17/IL-23 immunohistochemical detection could be employed to improve the identification of SS patients with a possible diagnosis in all cases that do not fully meet the American–European criteria for pSS, in particular when the germinal center is not present at histopathological analysis and anti-SSA and anti-SSB antibodies are undetectable in the serum [19]. Recently, a role for the synergetic interaction between IL-23 and TLR was demonstrated in SS; TLR2 ligation induces the production of IL-23 and IL-17 via IL-6, STAT3, and the NF-kB pathway in primary SS. Therefore, therapeutic strategies directed against the TLR/IL-17 pathway might be valid candidates for the treatment of SS [75]. In recent years, a thriving research sector has demonstrated that SS is often accompanied by fibrotic phenomena affecting the salivary glands mediated by the activation of the EMT program, triggered by the chronic inflammation that characterizes the disease [2,3]. Therefore, based on the attribution of an important role for IL-23 in the exacerbation of the disease, there are all the premises to identify a correlation between IL-23 expression, chronic inflammation, and fibrosis in SS.

### 5.5. Systemic Sclerosis

Systemic sclerosis (SSc) is a heterogeneous chronic, autoimmune, multisystem connective tissue disorder characterized by vasculopathy, inflammation, and progressive fibrosis of the skin and internal organs [76]. Interstitial lung disease (ILD) is a major common complication, along with pulmonary arterial hypertension, which is the leading cause of morbidity and mortality in scleroderma patients [77]. The finding of pulmonary fibrosis in patients with elevated IL-23 levels had a higher frequency when compared with subjects showing normal IL-23 levels [78]. The increased release of IL-23 showed a correlation with the initial stages of the disease and with the simultaneous presence of pulmonary fibrosis; it was not associated with other clinical manifestations of SSc [78]. IL-23 has been demonstrated to be abnormally expressed in autoimmunity, including Experimental autoimmune encephalomyelitis (EAE), collagen-induced arthritis, and inflammatory bowel disease [79]. In SSc, chronic T cell activation certainly occurs, contributing to the exacerbation of tissue inflammation [76]. Recent studies imply that IL-23 may determine the differentiation of activated T cells into effector T cells in SSc [78,80]. Based on these considerations, it is possible that Th17 cells, induced by IL-23, release IL-17, which can be implicated in molecular processes leading to vascular lesions, fibrosis, and autoimmunity in patients with SSc, exploiting the binding with the IL-17 receptor expressed on fibroblasts and endothelial cells [81]. Recently, a strong correlation between the onset of the disease, the initiation of pulmonary fibrosis, and the increase of IL-23 expression has been demonstrated, suggesting that Th17, stimulated by IL-23, is involved in the onset of SSc, but not in disease progression [78]. This hypothesis has also recently found radiological confirmation because, in SSc patients, there was a statistically significant difference as regards serum concentration of IL-23 in patients with pulmonary fibrosis by chest X-ray [82].

### 5.6. Multiple Sclerosis

MS is a chronic autoimmune disorder affecting an estimated two million people worldwide. The pathological hallmarks of MS include perivascular T-cell inflammation and disseminated demyelinating lesions [83]. Experimental autoimmune encephalomyelitis (EAE) is an inflammatory autoimmune pathology that can be induced in mice and, in addition, presents many similarities with human MS [79]. EAE is a complex disease in which the interaction between several immunopathological and neuropathological events determines an approximation of the pathological characteristics of MS manifested morphologically by inflammation, demyelination, axonal loss, and gliosis [79]. The main characteristic of the EAE condition is a fibrotic scar that determines an inhibitory environment hindering the remyelination; thus, anti-fibrotic drugs may serve as novel therapeutic targets for MS. As in MS, aberrant T lymphocytes traffic against the brain and spinal cord, causing disruption of the myelin sheath integrity of the central nervous system (CNS), leading to paresthesia, paraparesis, neuritis, and ataxia [84]. Because EAE presents clinical features similar to human MS, it could be used as a model to identify the clinical efficacy of targeting the IL-23 immune pathway. Indeed, specific anti-IL-23p19 antibodies were produced to test whether blocking the functionality of IL-23 reduced the clinical symptoms of EAE and whether it could also be used in human disease [85]. The treatment of anti-IL-23p19 diminishes the serum level of IL-17 as well as the expression of IFN-γ, IP-10, IL-17, IL-6, and TNF in the CNS, thus inhibiting multiple inflammatory signaling pathways that drive CNS autoimmune inflammation. In addition, the therapeutic efficacy of the anti-IL-23p19 antibody was demonstrated to prevent disease relapse [85]. Recently, the wealth of knowledge in this field has been enriched with new discoveries that have demonstrated IL-23 as a key factor driving inflammatory processes in the CNS [86]. In particular, a transgenic mouse with astrocyte-specific expression of IL-23 developed an ataxic phenotype and cerebellar infiltrates with high amounts of B lymphocytes. In these mice, in which EAE was induced, it was demonstrated that the local IL-23 production in the CNS determines the aggravation of the disease course with severe paraparesis and an ataxic phenotype, leading to the enhancement of gliosis and neuroinflammation in the CNS [86,87]. Certainly, further studies will be necessary to identify the mechanisms that explain the key role of IL-23 in MS, but the premises are very interesting, and the preliminary results are very intriguing.

## 6. New Therapeutic Challenges

### 6.1. IL-23 Blocking Agents

In the last two decades, several biological agents have been developed to ameliorate the knowledge of the interactions between the immune system and related cytokines, which affect the entire pathologic condition process. Some of these agents inhibit specific molecular pathways involved in the pathogenesis of autoimmune diseases, specifically in those characterized by severe fibrosis. Biological drugs targeting IL-23, either specifically (anti-p19) or in conjunction with IL-12 (anti-p40), have displayed a wide range of antagonistic activities because IL-23 is an important upstream regulator of pathways involved in fibrotic autoimmune diseases [88]. Since their approval, several real-life studies have been published on IL-23 inhibitors use in routine clinical practice, and real-life results of anti-IL-23 seem to confirm the promising findings of IL-23 demonstrated by clinical trials, highlighting the efficacy and safety profiles of this new class of biologic agents also in clinical practice [89]. Therefore, growing evidence supports the idea that drugs targeting IL-23 have shown promising efficacy in inflammatory bowel disease. Indeed, they were approved for the treatment of Crohn’s disease (CD) and, recently, for ulcerative colitis as well. Guselkumab, risankizumab, and tildrakizumab represent the latest biologic agents accepted for the treatment of psoriasis with varying degrees of severity and have ameliorated the perception of patients’ quality of life affected by fibrotic diseases [89]. In particular, Guselkumab, in which the mechanism of action occurs through selective inhibition of IL-23 via binding to its p19 subunit, demonstrated greater efficacy and durability of response in the treatment of plaque-type psoriasis compared with a placebo [90]. Therefore, emerging studies have observed a good therapeutic effect after treatment with Guselkumab of patients affected by psoriasis vulgaris complicated by SSc, improving each symptom of SSc, such as immune abnormalities, fibrosis, and vasculopathy [91]. Unfortunately, the administration of anti-IL-23 agents has not had positive effects in all patients, and they can cause undesirable immunological and non-immunological adverse events. However, these inhibitors tend to be well tolerated, with good safety profiles [92]. Ustekinumab, for example, already accepted for psoriatic arthritis, has shown promising data in SLE, but its evaluation in clinical trials has led to very contradictory results [93].

In conclusion, the recent evaluations on the therapeutic use of IL-23 seem to have two advantages: on the one hand, the possibility that IL-23 could represent a valid marker for the initial stages of autoimmune diseases, and, on the other, the possibility of identifying pharmacological treatments that specifically modulate the T helper’s immune response [94].

### 6.2. Epigenetics in the Control of IL-23 Expression in Autoimmune Diseases

Epigenetics studies how stress, age, and exposure to environmental factors, including physical and chemical agents, diet, and physical activity, can modify gene expression without modifying the DNA sequence. Epigenetic modifications of DNA regulate physiological processes, but recent data indicate that they play a role in the onset of diseases. A key factor in the development of autoimmunity is the impaired function of Treg cells. The identification and correction of diet, environmental, or stress factors improves the activity of Treg cells, and these considerations represent the epigenetic approach to autoimmune diseases [95]. Over the last few years, the field of epigenetics has been revolutionized by various innovative technologies aimed at determining these modifications (epimutations) through the analysis of DNA methylation, non-coding RNA expression, histone, and nucleosome modifications [95]. These data underline how the study of the genome and, even more, of the epigenome, helps to trace an individual profile that shows intra-individual variability. In this regard, epigenomics is also acquiring an important role in determining susceptibility to various diseases. The epigenetic study of IL-23 regulation, conducted in very recent times, has led to very intriguing results. The regulatory mechanism carried out via mTOR responsible for histone methylation mentioned above, e.g., in RA, represents an epigenetic modification that may regulate the IL-23-mediated process of EMT-dependent fibrosis [64]. In addition, Jin et al. report an epigenetic mechanism for the regulation of the gene Zranb1 (Zinc Finger RANBP2-Type Containing 1) responsible for the coding of the deubiquitinase Trabid, which seems to be involved in the regulation of IL-23 and correlated IL-12 expression in autoimmunity [96]. Deletion of Zranb1 in dendritic cells inhibits the expression of IL-12 and IL-23 by TLRs, impairing the differentiation of Treg and protecting mice from autoimmune inflammation. The role of Trabid takes place through the TLR-induced histone modifications at the IL-12 and IL-23 gene promoters, which involve deubiqutination. Another study conducted in SLE patients demonstrated the involvement of IL-23 in the STAT3-mediated alteration of the loci of RORγt at STAT binding sites, resulting in an exacerbated inflammatory function of Th17 in SLE [97]. Furthermore, evidence that an epigenetic mechanism involving TNF and the neural Wiskott–Aldrich syndrome protein (N-WASP) controls IL-23 expression in psoriatic keratinocytes has been provided by Li et al. [98]. Keratinocyte-restricted deletion of the N-WASP gene revealed an important function for N-WASP corresponding to increased TGF-β signaling, a potent pro-fibrotic cytokine. The loss of N-WASP in keratinocytes provokes IL-23 over-expression in keratinocytes, acting through the control of histone methylation mediated by IL-17 and TLRs. Once again, this evidence suggests that different independently studied pathways can converge in order to identify molecular bridges that can lead to a unique and complex mechanism of activation in autoimmune diseases.

Can epigenetics clarify which factors can determine the fibrotic evolution of autoimmune diseases? Or could epigenetics help identify common mechanisms that drive the chronic inflammation that characterizes multiple autoimmune diseases and that appears to be responsible for fibrosis? The answer seems to be far from being identified, but researchers engaged in this field of investigation are making giant strides and promising new perspectives for effective therapies.

## 7. Conclusions

Recent discoveries place IL-23 in a prominent position in the modulation of the immune response mediated by T helper lymphocytes. This has given rise to a series of investigations on the ability of IL-23 to modulate autoimmune processes characterized by chronic inflammation, which, frequently, evolve towards fibrotic processes. Although the mechanisms underlying the pro-fibrotic activity of IL-23 are not clear, some underlying mechanisms common to several fibrotic and autoimmune diseases have been identified. This suggests that we are on the right track and that, soon, therapies that block the activity or the release of IL-23 could represent a valid therapeutic alternative in the course of autoimmune fibrotic diseases.

## Data Availability

Not applicable.
